# Smartphone fundoscopy with 20 dioptres lens: our experience

**DOI:** 10.22336/rjo.2024.27

**Published:** 2024

**Authors:** David-Ionuț Beuran, Cătălin Cornăcel, Călin Petru Tătaru

**Affiliations:** * Department of Ophthalmology, “Dr. Carol Davila” Central Military University Emergency Hospital, Bucharest, Romania; ** Clinical Department of Ophthalmology, “Carol Davila” University of Medicine and Pharmacy, Bucharest, Romania; *** Department of Ophthalmology, Clinical Hospital for Ophthalmological Emergencies, Bucharest, Romania

**Keywords:** smartphone fundoscopy, papilledema, bedside examination

## Abstract

**Objective:** Assessment of the utility of smartphone fundoscopy in diagnosing posterior pole pathologies.

**Methods:** An iPhone 12 and a 20D Volk lens were used for smartphone fundoscopy. Patients needing bedside consultation were examined with direct ophthalmoscopy and smartphone fundoscopy. Some patients were examined with this technique after slit lamp examination.

**Results:** Over one year 23 bedside fundus examinations were performed and 2 papilledema were diagnosed. After initial slit lamp examination, photos of various pathologies were taken: age-related macular degeneration, branch retinal artery occlusion, arterial embolus, branch retinal vein occlusion, non-arteritic anterior ischemic optic neuropathy, myelinated retinal nerve fiber layer, choroidal naevus.

**Discussion:** With the 20D lens, the image is overturned, magnified 3,13X, and the field of view is 46°. The utility was demonstrated in literature by teaching students this technique and using it in screening for retinopathy of prematurity. The weighted retinal irradiance was measured in two studies. It was 4,6 mW/cm2 in one and from 0,58 to 2,30 mW/cm2 in the other, within safe limits.

**Conclusions:** Smartphone fundoscopy is a fast, accessible, and safe technique for fundus examinations. Other departments could use it for the diagnosis of papilledema.

## Introduction

At the beginning of the last decade, several authors described a new technique for retinal examination. It consisted of using the light source of a phone and a 20 dioptres lens. With those two, a system working as an indirect ophthalmoscope is made and digital images of the fundus are captured in the phone camera [**1**,**2**].

Nowadays, smartphones are ubiquitous, and almost all have a high-quality camera that makes this technique a good alternative when a slit lamp and direct or indirect ophthalmoscope are not available. Furthermore, it is a useful and cheap option for fundus photography.

## Materials and methods 

Over a year, all patients needing bedside consultation were examined with a direct ophthalmoscope and a smartphone fundoscopy. An iPhone 12 and a 20D Volk lens were used for smartphone fundoscopy. 

Some patients were examined with this technique after slit lamp examination.

For smartphone fundoscopy, the following steps must be followed:

1. The anterior chamber depth should be checked. A direct ophthalmoscope with settings for the anterior pole (i.e. +10 dioptres) is a good option for bedside consultations.

2. Intraocular pressure should be checked. For bedside consultations, the digital measure of intraocular pressure can be used.

3. If the anterior chamber depth is not small and the eye has a normal tonus, drops should be applied to dilate the pupil. 

4. Phone settings: the phone is accessed in video mode; the optical zoom is set at 2X or 3X and the flash is turned on. When starting recording, the flash must remain activated. To do that filming should be started in a dark place (e.g. the camera should be covered with a hand and filming should be started). 

5. The 20D lens should be taken in the non-dominant hand and the phone with camera in the dominant hand. The lens should be held between the thumb and index and the hand should be stabilized by putting the medius finger on the eyebrow of the examined eye. The lens should be kept at a distance of about 5-8 cm from the eye. 

6. The patient should look straight ahead. The light from the phone must be perpendicular to the lens. Together with the eye, those three components must be on the same axis. The phone should be kept approximately 30 cm away from the lens. 

7. The patient should be asked to look right, up, left, and down for the retinal periphery.

8. After examination, the recording should be stopped. 

9. The movie should be watched and stopped when a good image appears and the fundus photo should be screenshot. 

A similar protocol was described by Nazari et al. in 2017 [**3**]. 

Patients were hospitalized at “Dr. Carol Davila” Central Military University Emergency Hospital, Bucharest. 

## Results

**Fig. 1:**
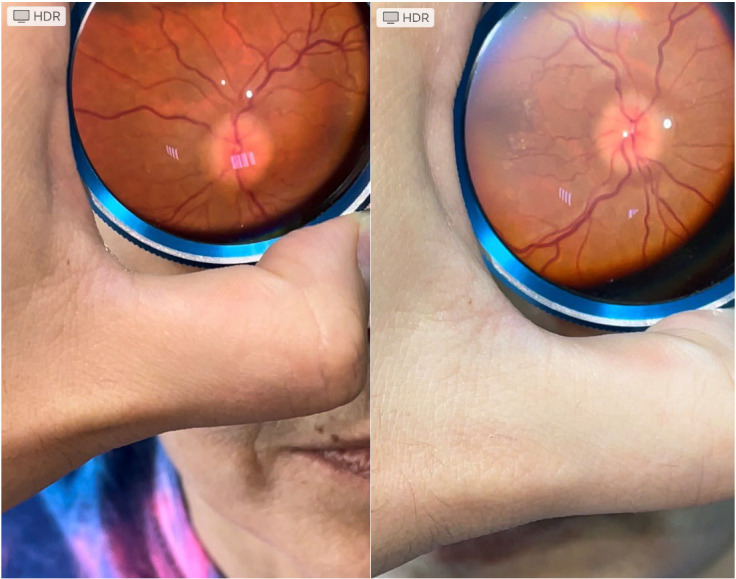
Papilledema, the right and left eye of the same patient

Over one year, 23 bedside examinations were made: emergency room - 1 patient, neurosurgery department - 2 patients, neurology department - 20 patients. Two papilledema were diagnosed (**Fig. 1**). 

**Fig. 2:**
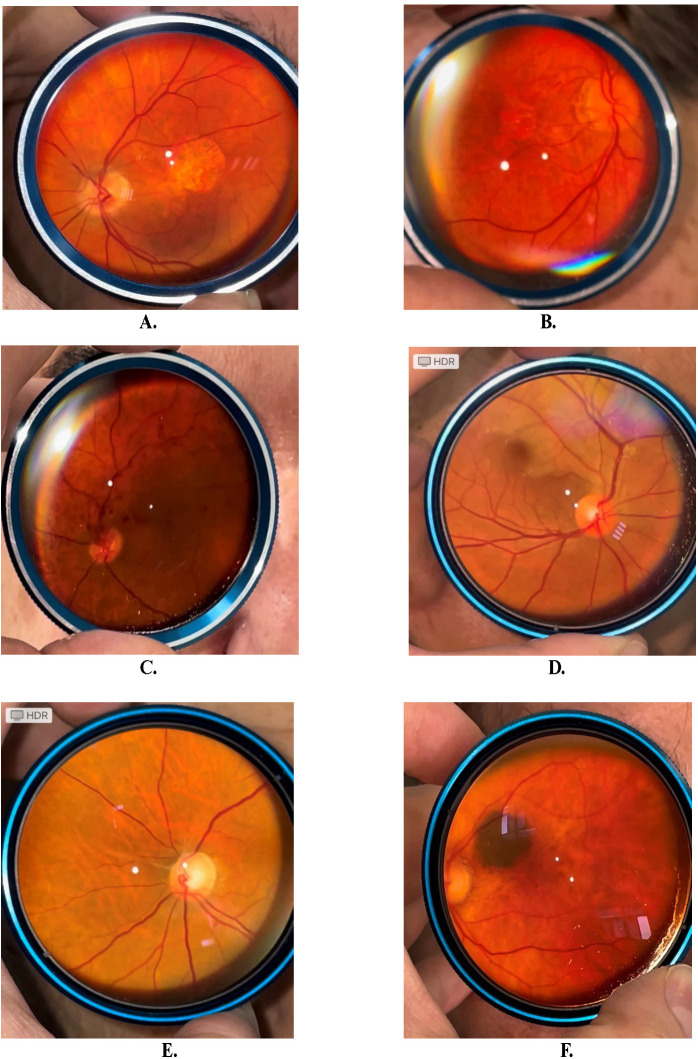
Fundus photos from different patients: dry age-related macular degeneration, geographic atrophy (**A, B**), branch retinal vein occlusion (**C**), acute (**D**) and old (**E**) branch retinal arterial occlusion, choroidal naevus (**F**)

Furthermore, fundus photos were taken after the initial diagnosis with slit lamp and 90D lens. The following pathologies were observed: age-related macular degeneration (three wet, and two dry (**Fig. 2 A, B**), a total of five eyes), branch retinal vein occlusion (one eye - **Fig. 2 C**), branch retinal artery occlusion (two eyes - **Fig. 2 D, E**), arterial embolus (two eyes), non-arteritic anterior ischemic optic neuropathy (one eye), myelinated retinal nerve fiber layer (one eye), choroidal naevus (two eyes - **Fig. 2 F**).

## Discussion

*Technique*

With the 20D lens, the image is magnified 3,13X, and the field of view is 46°. The magnification and field of view vary depending on the power of the lens. The working distance from the cornea varies similarly, and for a 20D lens is about 5 cm. 20D lens is the most commonly used lens [**4**]. Regardless of the lens power, the obtained image is overturned.

*Utility*

One hundred thirty-seven second-year medical students received instructions on direct ophthalmoscopy and smartphone fundoscopy. Almost a quarter of them (24%) preferred smartphone fundoscopy over direct ophthalmoscopy. In conclusion, smartphone fundoscopy was a non-inferior technique to direct ophthalmoscopy in identifying structures and teaching it should be considered [**5**]. 

A study described an iPhone use combined with a 20D lens in screening for retinopathy of prematurity in Lagos, Nigeria. The images captured were satisfactory for staging and determining the need for treatment. In conclusion, the technique is useful for screening in resource-poor conditions [**6**]. 

*Safety*

The weighted retinal irradiance for smartphone fundoscopy was measured in a study. It was 4,6 mW/cm2, 150 times below the thermal limit of 706 mW/cm2. Furthermore, compared to a standard indirect ophthalmoscope, the levels observed were 46 mW/cm2, 10 times bigger than with a smartphone. The phone tested was the iPhone 4 [**7**].

In another study, the spectrum of the light-emitting diode flashlight of all tested smartphones was in the optically safe visible spectrum between 400 and 750 nanometres. Weighted irradiance was within the safe limits, from 0,58 to 2,30 mW/cm2, without measurable thermal effect. In conclusion, smartphone fundoscopy seemed within safe limits [**8**].

## Conclusions

Smartphone fundoscopy with 20 dioptres lens is an easy, fast, and safe technique for fundus examination. Because it is an accessible alternative to direct ophthalmoscopy, other departments could use it to diagnose papilledema. Furthermore, it could be used for teaching students, additional patient explanations, and in telemedicine. 

**Conflict of Interest Statement**

The authors state no conflict of interest. 

**Informed Consent and Human and Animal Rights Statement**

Informed consent has been obtained from the patients included in the study. 

**Authorization for the use of human subjects**

Not applicable.

**Acknowledgments**

None. 

**Sources of Funding**

None.

**Disclosures**

None. 
